# Supraventricular arrhythmia, N-terminal pro-brain natriuretic peptide and troponin T concentration in relation to incidence of atrial fibrillation: a prospective cohort study

**DOI:** 10.1186/s12872-021-01942-6

**Published:** 2021-03-12

**Authors:** Jun Xiao, Anders P. Persson, Gunnar Engström, Linda S. B. Johnson

**Affiliations:** 1 Department of Cardiovascular Surgery, Fujian Medical University Union Hospital, Fuzhou, China; 2 Department of Clinical Sciences in Malmö, Lund University, CRC 60:13, Jan Waldenströms gata 35, 205 02 Malmö, Sweden

**Keywords:** Atrial fibrillation, Supraventricular extrasystole, Premature atrial contractions, Supraventricular tachycardia, NT-proBNP, Troponin T

## Abstract

**Background:**

Frequent supraventricular arrhythmia is associated with increased incidence of atrial fibrillation. However, it is unknown whether the prognostic significance of supraventricular arrhythmia is modified by plasma levels of N-terminal pro-brain natriuretic peptide (NT-proBNP) or troponin T (TnT). This study examined the interrelationships between NT-proBNP, TnT levels and frequent supraventricular arrhythmia, and whether these biomarkers and a measure of frequent supraventricular arrhythmia could improve risk assessment for incidence of AF.

**Methods:**

Supraventricular extrasystoles (SVEs) and supraventricular tachycardias were assessed from 24-h electrocardiograph recordings in 373 individuals initially without AF. Elevated NT-pro-BNP, TnT and SVEs was defined as a measurement in the top quartile of the study population distribution. Incident cases of AF were retrieved by linkage with the Swedish National Patient Register.

**Results:**

During a mean follow-up of 15.4 years, 88 subjects had a diagnosis of AF. After multivariable adjustment, individuals with both elevated NT-proBNP and frequent SVEs had a significantly increased incidence of AF, compared to subjects without elevated NT-proBNP or frequent SVEs (hazard ratio (HR) 4.61, 95% confidence interval (CI) 2.45–8.69), and compared to individuals with either elevated NT-proBNP or frequent SVEs (both *P* < 0.05). HRs for frequent SVEs alone or elevated NT-proBNP alone were 2.32 (95% CI 1.33–4.06) and 1.52 (95% CI 0.76–3.05), respectively. The addition of NT-pro-BNP and SVEs to a validated risk prediction score for AF, CHARGE-AF, resulted in improved prediction (Harrell’s C 0.751 (95% CI 0.702–0.799) vs 0.720 (95% CI 0.669–0.771), *P* = 0.015).

**Conclusion:**

Subjects with both elevated NT-proBNP and frequent SVEs have substantially increased risk of AF, and the use of these variables could improve long-term prediction of incident AF.

**Supplementary Information:**

The online version contains supplementary material available at 10.1186/s12872-021-01942-6.

## Introduction

Atrial fibrillation (AF) is the most common sustained arrhythmia, especially among the elderly, and is associated with increased mortality and morbidity [1–3]. Improved risk assessment and early identification of individuals with high risk of AF could improve stroke prevention and lead to important public health benefits. Even though there are currently no validated prevention programs for AF, recent research indicates that early stages of the disease could be reversible [4, 5]. Furthermore, since AF can be asymptomatic, stroke events associated with AF may occur before AF is diagnosed. If individuals with high AF risk could be identified, this could enable repeated or prolonged electrocardiogram (ECG) monitoring, and early anticoagulation in order to prevent stroke.

Although the pathophysiology of AF is complex, considerable advances have been made in understanding the underlying pathophysiological mechanisms. There is convincing evidence that atrial myopathy, a state of atrial structural and electrical remodeling, precedes the development of AF [6, 7]. Structural remodeling includes atrial dilation and myocardial damage. This may result in elevated N-terminal pro-brain natriuretic peptide (NT-proBNP) and troponin T (TnT) [8, 9], both of which have been linked to a greater risk of AF [10, 11]. Electrical remodeling, on the other hand, can be measured by increased frequency of supraventricular extrasystoles (SVEs) and supraventricular tachycardias (SVTs), which are also highly related to AF risk [12–16]. It is plausible, therefore, that subjects with both elevated NT-pro-BNP or TnT and frequent supraventricular extrasystoles could have substantially increased risk of AF.

The CHARGE-AF score is a well validated risk score for prediction of AF, and a simplified form of the score can be calculated from clinically available parameters [17, 18]. The aim of this study was to examine whether the predictive value for incident AF is improved by combining SVEs and NT-proBNP or TnT levels, and if these risk factors add predictive information on top of the validated CHARGE-AF risk score for AF.

## Methods

### Study population

The study population was derived from the population-based, prospective Malmö Diet and Cancer Study cohort, which has been described elsewhere [16], and which consists of 30,446 individuals (age range 44–73 years, 39.8% men) recruited during the period of 1991–1996. A subsample consisting of 6103 individuals were randomly invited to receive additional screening in a cardiovascular sub-study. In 1998–2000, a re-examination was performed for 909 individuals from the cardiovascular sub-study. These individuals were randomly invited after stratification for degree of insulin resistance, as measured by the homeostasis model assessment for insulin resistance (HOMA-IR, calculated as fasting insulin (mIU/l) fasting blood glucose (mmol/l)/22.5)). High HOMA-IR was slightly oversampled (15% from each of quartiles 1 and 2, 30% from quartile 3, and 40% from quartile 4). Of the 909 individuals, a random subsample of 388 individuals underwent 24hECG screening (age range 53–74, 45.6% men). The study conforms to the declaration of Helsinki. Subjects with inadequate 24hECG recordings (n = 5), a diagnosis of AF (n = 6), missing NT-proBNP and TnT results (n = 4) were excluded; resulting in a final study population consisting of 373 subjects in total. Due to missing data for HOMA-IR score (n = 7), low density lipoprotein (LDL) (n = 1), and height and weight (n = 1), multivariable models included 364 subjects (Additional file 1: Supplementary Figure 1).

### Data collection

Physical examination was performed for all subjects at baseline. Height and weight were measured standing in light indoor clothing, using a fixed stadiometer and a balance beam scale. Blood pressure was measured after 10 min supine rest, using a sphygmomanometer with a modifiable cuff. All patients completed an extensive self-administered questionnaire, from which current smoking status was retrieved. History of diabetes, coronary events, and heart failure were obtained through a combination of information from questionnaires and data from hospital registers. Blood samples were drawn after overnight fast and analyzed using standard laboratory procedures at the Malmö University Hospital. The samples were stored at − 70 °C. Plasma samples were analyzed by electrochemiluminescence using R-PLEX human NT-proBNP and R-PLEX human TnT (cardiac) assays (Meso Scale Discovery, Gaithersburg, MD) on a Sector S600 instrument at the Clinical Biomarkers Facility, SciLifeLab, Uppsala, Sweden. The NT-proBNP assay was run at a 1:10 dilution (average dynamic range 1.1 pg/ml–10,000 pg/ml) whereas Troponin T was run neat (average dynamic range 4.4 pg/ml to 500 pg/ml).

### 24-h ECG

All patients underwent a 24-h 3-lead (X, Y, Z coupling) Holter ECG recording, with 256-Hz sampling rate, for arrhythmia detection, which has been described before [16]. The equipment used was Life card CF digital Holter recorder with 12-bit resolution (Spacelabs Health care, Issaquah, WA). Findings were classified using the Pathfinder SL analysis tool (Spacelabs Healthcare). All ECG recordings were manually verified. Mean analysis time was 23.2 h, with a standard deviation (SD) of 1.2 h. SVEs was defined as a premature narrow complex beat preceded by a normal sinus beat. Three or more consecutive SVEs with a heart rate ≥ 100 beats per minute was defined as a SVT.

### CHARGE-AF risk score

The CHARGE-AF risk score was calculated for each individual according to previously described specifications [18, 19]. Briefly, the score is calculated  as follows: 0.508 × age (5 years) + 0.248 × height (10 cm) + 0.115 × weight (15 kg) + 0.197 × systolic blood pressure (20 mm Hg) − 0.101 × diastolic blood pressure (10 mm Hg) + 0.359 × current smoker + 0.349 × antihypertensive medication + 0.237 × diabetes + 0.701 × congestive heart failure + 0.496 × myocardial infarction. In the present study, age, height, weight, systolic and diastolic blood pressure, current smoking status, antihypertensive medication, diabetes, history of heart failure, and history of coronary event (including coronary artery bypass grafting surgery and percutaneous coronary intervention) at baseline were used in the CHARGE-AF risk score calculations.

### End-point ascertainment

Subjects were followed until first AF diagnosis (diagnosis codes 427D for the 9th revision of the International Classification of Diseases (ICD-9), and I48 for the 10th revision (ICD-10)), death, emigration, or end of the study (December 31, 2018), whichever came first. Atrial flutter was not distinguished from AF, due to the similarities between these diagnoses [20]. All endpoints were retrieved from the National Patient Register administered by the Swedish National Board of Health and Welfare, which covers all inpatient diagnoses since the year 1987 and all hospital outpatient diagnoses since the year 2000. A validation study of the AF cases in the Malmö Diet and Cancer study showed an accuracy of 95% [21]. The conventional duration of AF for diagnosis is 30 s in Sweden. The regional ethics review board in Lund has approved the study (LU 51/90).

### Statistical methods

All analyses were performed using SPSS 25.0 and R 3.6.3. Negative binominal regression was used to estimate the relation between the numbers of SVEs per 24 h or SVTs per 24 h and per quartile increment in the NT-proBNP or TnT concentrations. Cox regression analyses were used to study the association between the combination of supraventricular arrhythmias and plasma biomarkers and incidence of AF. Elevated SVEs, SVTs, NT-proBNP, and TnT was defined as a measurement in the top quartile, corresponding to > 129.18 SVEs/24 h, 3.06 SVTs/24 h, 32.80 pg/ml NT-proBNP and > 1.38 pg/ml TnT. Elevated supraventricular activity (SVA) was defined as either the top quartile of SVEs or SVTs. Based on previous studies [16, 22], potential confounders which were related to both exposure and outcome were identified, and the following two pre-specified models were used. *Model 1* adjusted for age and sex, and *Model 2* additionally adjusted for current smoking, use of anti-hypertension medications, systolic blood pressure, LDL, natural log of HOMA-IR, height, and weight. A *P*-value below 0.05 denoted statistical significance.

The log-rank test was used to compare the cumulative incidence between combinations of NT-proBNP and SVEs, stratified by elevated CHARGE-AF-score (top tertile) or not (tertile 1,2). Performance of Cox regression models for AF was assessed using C-statistic. Differences in C-statistics between a model with CHARGE-AF alone and models with additional risk factors were compared by Student t test [23]. Furthermore, category free net reclassification improvement (NRI) was calculated for 15-year AF risk prediction to compare the predictive performance of the CHARGE-AF score model to models combining the CHARGE-AF score with measures of SVEs and biomarkers.

## Results

### Baseline characteristics

The mean age at baseline was 64.0 ± 5.9 years. Of the 373 included individuals, 203 (54.4%) were women. At baseline 22.8% (n = 85) of study participants were smokers. Information of baseline characteristics is presented in Table 1.

**Table 1:** Baseline characteristics

	All (n = 373)	Men (n = 170)	Women (n = 203)
Case of AF (n, %)	88 (23.6)	46 (27.1)	42 (20.7)
Age (years)	64.0 (5.9)	64.1 (6.1)	64.0 (5.7)
Current smoking (n, %)	85 (22.8)	45 (26.5)	40 (19.7)
History of diabetes (n, %)	32 (8.6)	18 (10.6)	14 (6.9)
History of coronary events (n, %)	8 (2.1)	5 (2.9)	3 (1.5)
History of heart failure (n, %)	2 (0.5)	1 (0.6)	1 (0.5)
Anti-hypertensive medication (n, %)	95 (25.5)	38 (22.4)	57 (28.1)
Systolic blood pressure (mmHg)	143.6 (18.5)	145.2 (18.6)	142.3 (18.3)
Diastolic blood pressure (mmHg)	88.3 (9.6)	90.3 (9.6)	86.6 (9.3)
Height (cm; n = 372)	169.0 (9.4)	176.3 (6.9)	162.8 (6.3)
Weight (kg; n = 372)	77.3 (13.2)	83.9 (12.2)	71.8 (11.4)
HOMA-IR (n = 366)	2.54 (1.83)	2.75 (2.03)	2.36 (1.64)
LDL (mmol/l; n = 372)	3.72 (0.83)	3.67 (0.76)	3.76 (0.89)
NT-proBNP (pg/ml)	41.84 (100.73)	53.22 (128.35)	32.32 (68.56)
Troponin T (pg/ml)	1.14 (1.40)	1.46 (1.66)	0.88 (1.07)
CHARGE-AF risk score (n = 372)	12.01 (0.75)	12.26 (0.73)	11.81 (0.71)

Values are presented as mean (SD) or n (%)

*AF* atrial fibrillation, *HOMA-IR* homeostasis model assessment of insulin resistance, *LDL* low-density lipoprotein, *NT-proBNP* N terminal pro B type natriuretic peptide

### Plasma biomarkers, supraventricular arrhythmias and incidence of AF

**Fig. 1:**
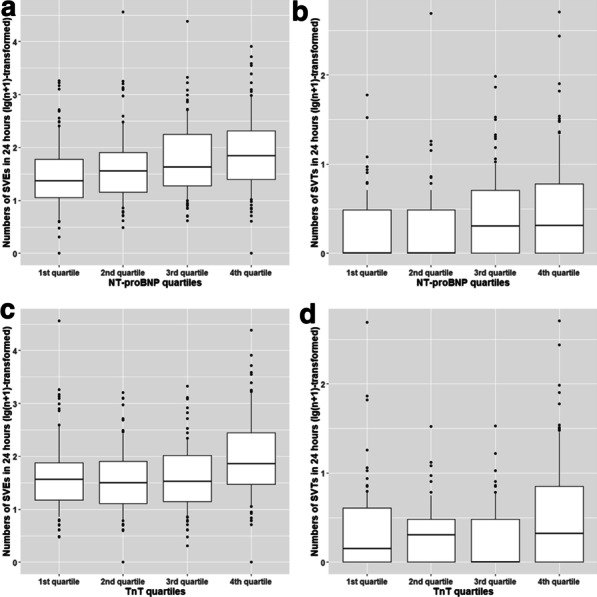
The relation between SVEs/SVTs in 24 h and quartiles of NT-proBNP/TnT. The numbers of SVEs and SVTs were transformed by lg(n + 1). **a** SVEs/24 h and NT-proBNP quartiles. **b** SVTs/24 h and NT-proBNP quartiles. **c** SVEs/24 h and TnT quartiles. **d** SVTs/24 h and TnT quartiles. The boxes illustrate median, 25th and 75th percentiles. Vertical lines include the 10th and 90th percentiles. *Abbreviations NT-proBNP* N terminal pro B type natriuretic peptide, *SVEs* supraventricular extrasystoles, *SVTs* supraventricular tachycardias, *TnT* troponin T

During a mean follow-up time of 15.4 years, 88 subjects were diagnosed with AF (cumulative incidence 23.6%). The number of SVEs and SVTs in 24 h were positively associated with quartiles of NT-proBNP and TnT concentrations (Fig. 1, Additional file 1: Supplementary Table 1). After multivariable adjustment NT-proBNP and TnT were related to the number of SVEs in 24 h (Odds ratio (OR)_NT-proBNP_ = 1.64, 95% confidential interval (CI) 1.33–2.02; OR_TnT_ = 1.33, 95% CI 1.10–1.60, both per quartile increase; Additional file 1: Supplementary Table 1). A similar association was observed for SVTs in 24 h: OR_NT-proBNP_ = 1.60, 95% CI 1.27–2.02; OR_TnT_ = 1.33, 95% CI 1.03–1.62 per quartile increase (Additional file 1: Supplementary Table 1).

The association between the combinations of NT-proBNP and supraventricular arrhythmias with risk of AF is reported in Table 2. After multivariable adjustment individuals with both elevated NT-proBNP and elevated SVEs had the highest risk of incident AF, compared to subjects with normal values for both SVEs and NT-pro-BNP, and compared to subjects with either elevated SVEs or elevated NT-pro-BNP, for which groups an intermediate risk was observed. Similar results were found for combinations of NT-proBNP and SVTs, as well as NT-proBNP and SVA.

**Table 2:** Incidence of AF by occurrence of elevated NT-proBNP and frequent supraventricular arrhythmias

N = 373	Subjects	Incident AF cases	Incidence rate (per 1000 person-years)	Model 1 h (95%CI)	Model 2 h (95%CI)
**NT-proBNP and SVEs**					
Non-elevated NT-proBNP, infrequent SVEs	220	35	9.49	Ref	Ref
Elevated NT-proBNP, infrequent SVEs	59	12	15.07	1.45 (0.75, 2.80)^†^	1.52 (0.76, 3.05)^†^
Non-elevated NT-proBNP, frequent SVEs	60	22	25.30	**2.48 (1.45, 4.26)**	**2.32 (1.33, 4.06)**^†^
Elevated NT-proBNP, frequent SVEs	34	19	47.05	**4.19 (2.30, 7.65)**	**4.61 (2.45, 8.69)**
Interaction NT-proBNP * SVEs				*P* = 0.740	*P* = 0.582
**NT-proBNP and SVTs**					
Non-elevated NT-proBNP, infrequent SVTs	214	31	8.68	Ref	Ref
Elevated NT-proBNP, infrequent SVTs	61	16	20.24	**1.99 (1.08, 3.67)**	**2.03 (1.07, 3.87)**^‡^
Non-elevated NT-proBNP, frequent SVTs	66	26	26.08	**2.52 (1.48, 4.29)**	**2.26 (1.30, 3.95)**
Elevated NT-proBNP, frequent SVTs	32	15	36.66	**3.55 (1.88, 6.70)**	**4.46 (2.24, 8.88)**
Interaction NT-proBNP *SVTs				*P* = 0.443	*P* = 0.945
**NT-proBNP and SVA**					
Non-elevated NT-proBNP, non-elevated SVA	195	28	8.54	Ref	Ref
Elevated NT-proBNP, non-elevated SVA	53	12	17.11	1.74 (0.88, 3.44)^§^	1.76 (0.86, 3.62)^§^
Non-elevated NT-proBNP, elevated SVA	85	29	22.63	**2.23 (1.32, 3.79)**	**2.10 (1.21, 3.65)**^§^
Elevated NT-proBNP, elevated SVA	40	19	38.10	**3.65 (1.98, 6.73)**	**4.33 (2.26, 8.31)**
Interaction NT-proBNP *SVA				*P* = 0.894	*P* = 0.740

Model 1: age, sex adjusted (n = 373)

Model 2: Model 1 + smoking status + anti-hypertension medications + systolic blood pressure + LDL + log of HOMA-IR + height + weight (n = 364)

Frequent SVEs, SVTs, and elevated NT-proBNP were defined as the top quartile, corresponding to > 129.18 SVEs/24 h, 3.06 SVTs/24 h and 32.80 pg/ml NT-proBNP, respectively; elevated SVA was defined as frequent SVEs or SVTs

*AF* atrial fibrillation, *NT-proBNP* N terminal pro B type natriuretic peptide, *SVEs* supraventricular extrasystoles, *SVTs* supraventricular tachycardias, *SVA* supraventricular activity

^†^*P* < 0.05 when compared to group with elevated NT-proBNP and frequent SVEs

^‡^*P* < 0.05 when compared to group with elevated NT-proBNP and frequent SVTs

^§^*P* < 0.05 when compared to group with elevated NT-proBNP and elevated SVA

Results for the association of combinations of TnT and supraventricular arrhythmias with incident AF are presented in Table 3. The highest risk of AF was found among individuals with both elevated TnT and supraventricular arrhythmias compared to subjects without.

**Table 3:** Incidence of AF by occurrence of elevated TnT and frequent supraventricular arrhythmias

N = 373	Subjects	Incident AF cases	Incidence rate (per 1000 person-years)	Model 1 h (95%CI)	Model 2 h (95%CI)
**TnT and SVEs**					
Non-elevated TnT, infrequent SVEs	225	37	10.00	Ref	Ref
Elevated TnT, infrequent SVEs	54	10	12.76	1.09 (0.54, 2.20)^†^	0.96 (0.47, 1.97)^†^
Non-elevated TnT, frequent SVEs	55	19	23.69	**2.24 (1.28, 3.92)**	**2.30 (1.30, 4.09)**
Elevated TnT, frequent SVEs	39	22	46.69	**3.71 (2.11, 6.51)**	**3.07 (1.74, 5.41)**
Interaction TnT *SVEs				*P* = 0.377	*P* = 0.501
**TnT and SVTs**					
Non-elevated TnT, infrequent SVTs	221	33	9.18	Ref	Ref
Elevated TnT, infrequent SVTs	54	14	18.22	1.66 (0.88, 3.12)	1.43 (0.75, 2.74)
Non-elevated TnT, frequent SVTs	59	23	25.27	**2.40 (1.40, 4.11)**	**2.29 (1.31, 4.02)**
Elevated TnT, frequent SVTs	39	18	37.02	**2.99 (1.65, 5.45)**	**2.58 (1.40, 4.76)**
Interaction TnT *SVTs				*P* = 0.528	*P* = 0.599
**TnT and SVA**					
Non-elevated TnT, non-elevated SVA	198	30	9.22	Ref	Ref
Elevated TnT, non-elevated SVA	50	10	13.79	1.27 (0.62, 2.62)^‡^	1.07 (0.51, 2.24)^‡^
Non-elevated TnT, elevated SVA	82	26	20.79	**1.97 (1.16, 3.35)**	**1.91 (1.10, 3.33)**
Elevated TnT, elevated SVA	43	22	41.55	**3.35 (1.87, 5.99)**	**2.85 (1.58, 5.13)**
Interaction TnT *SVA				*P* = 0.533	*P* = 0.493

Model 1: age, sex adjusted (n = 373)

Model 2: Model 1 + smoking status + anti-hypertension medications + systolic blood pressure + LDL + natural log of HOMA-IR + height + weight (n = 364)

Frequent SVEs, SVTs, and elevated TnT were defined as the top quartile, corresponding to > 129.18 SVEs/24 h, 3.06 SVTs/24 h and > 1.38 pg/ml TnT, respectively; elevated SVA was defined as frequent SVEs or SVTs

*AF* atrial fibrillation, *TnT* troponin T, *SVEs* supraventricular extrasystoles, *SVTs* supraventricular tachycardias, *SVA* supraventricular activity

^†^*P* < 0.05 when compared to group with elevated TnT and frequent SVEs

^‡^*P* < 0.05 when compared to group with elevated TnT and elevated SVA

### NT-proBNP, SVEs, and CHARGE-AF score

**Fig. 2:**
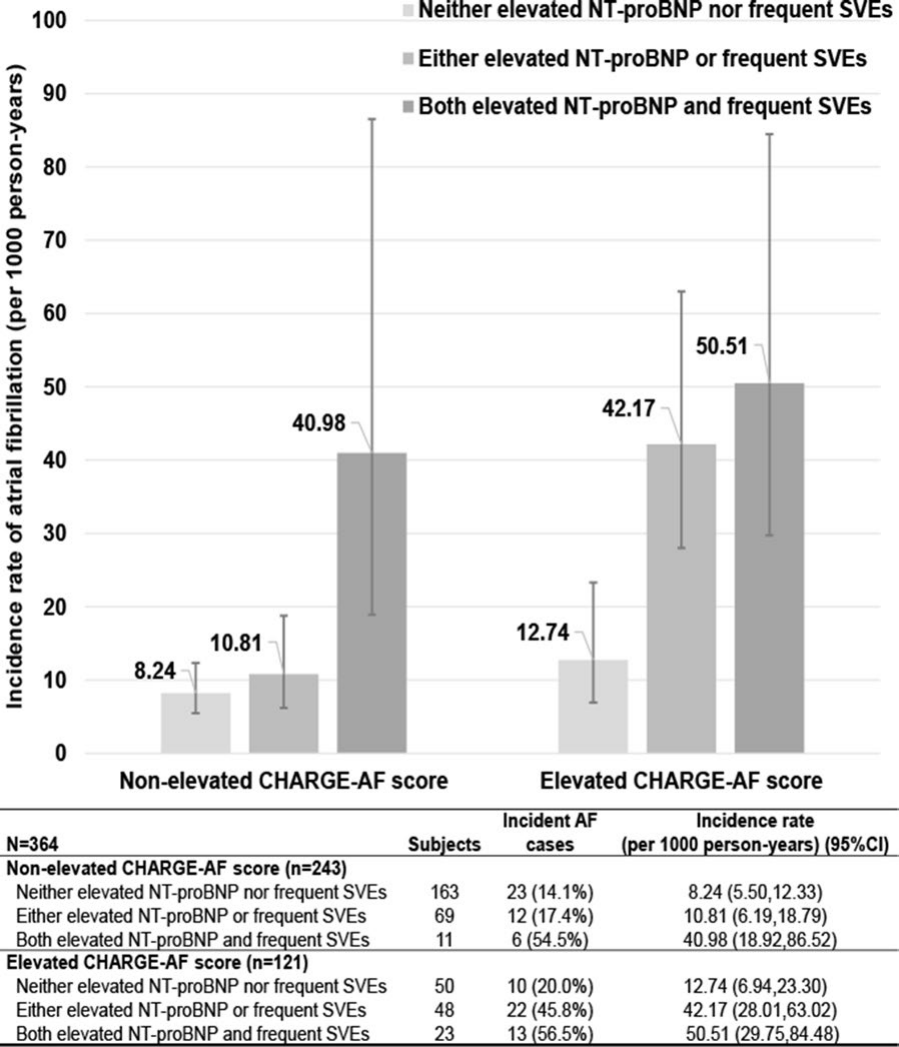
Incidence rates (95% confidence intervals) of AF in relation to a combination of elevated NT-proBNP and frequent SVEs stratified by elevated CHARGE-AF score. Frequent SVEs and elevated NT-proBNP were defined as the top quartile, corresponding to > 129.18 SVEs/24 h and 32.80 pg/ml NT-proBNP, respectively; elevated CHARGE-AF score was defined as top tertile. *Abbreviations SVEs* supraventricular extrasystoles, *NT-proBNP* N terminal pro B type natriuretic peptide

The relationship between NT-pro-BNP, SVEs and incidence of AF was also analyzed for elevated and normal levels of CHARGE-AF score. The combination of both elevated NT-pro-BNP and frequent SVEs was more common among subjects with an elevated CHARGE-AF score; 4.5% in the lower two tertiles compared to 19.0% in the top tertile (chi^2^ test value = 20.005, *P* < 0.001). Incidence of AF in individuals with high CHARGE-AF and both elevated NT-proBNP and elevated SVEs was 50.5 per 1000 as compared to 12.7 per 1000 for those with high CHARGE-AF score and normal SVEs and NT-proBNP (Fig. 2 and Additional file 1: Supplementary Figure 2).

The CHARGE-AF risk score had good discrimination for incident AF (C-statistic 0.720, 95% CI 0.669–0.771; Table 4). The highest C-statistic was found for a model including CHARGE-AF risk score, elevated NT-proBNP, and frequent SVEs: 0.751 (95% CI 0.702–0.799); this was significantly higher than CHARGE-AF score alone (*P* = 0.015; Table 4). Similarly, the category-free NRI also showed significant improvements in prediction of AF when elevated NT-proBNP and frequent SVEs were simultaneously added on top of CHARGE-AF score (NRI = 0.300, 95% CI 0.166–0.452; Table 4).

**Table 4:** C-statistics and category free net reclassification improvement (NRI) of predictive models for incident AF^†^

N = 364	C-statistic (95%CI)	P for difference^‡^	NRI
CHARGE-AF score	0.720 (0.669, 0.771)	Reference	Reference
Frequent SVEs	0.778 (0.703, 0.854)	0.075	− 0.222 (− 0.372, 0.059)
Elevated NT-proBNP	0.726 (0.635, 0.817)	0.448	− **0.277 (**− **0.427,** − **0.001)**
Elevated TnT	0.705 (0.610, 0.799)	0.376	− **0.258 (**− **0.425,** − **0.098)**
Frequent SVEs + elevated NT-proBNP	0.768 (0.704, 0.832)	0.084	− 0.126 (− 0.334, 0.158)
CHARGE-AF score + frequent SVEs	**0.743 (0.694, 0.793)**	**0.020**	**0.311 (0.182, 0.440)**
CHARGE-AF score + elevated NT-proBNP	0.734 (0.685, 0.783)	0.110	0.288 (− 0.028, 0.385)
CHARGE-AF score + elevated TnT	0.717 (0.664, 0.769)	0.392	0.201 (− 0.176, 0.362)
CHARGE-AF score + frequent SVEs + elevated NT-proBNP	**0.751 (0.702, 0.799)**	**0.015**	**0.300 (0.166, 0.452)**

Frequent SVEs, elevated NT-proBNP and elevated TnT were defined as the top quartile, corresponding to > 129.18 SVEs/24 h, 32.80 pg/ml NT-proBNP and > 1.38 pg/ml TnT, respectively

*AF* atrial fibrillation, *SVEs* supraventricular extrasystoles, *NT-proBNP* N terminal pro B type natriuretic peptide; *TnT* troponin T

^†^Net reclassification improvement was calculated for a 15-year AF risk prediction

^‡^*P* for difference in C-statistics compared to the Reference group

## Discussion

Findings from the current study suggest that subjects with both elevated NT-pro-BNP or TnT and frequent supraventricular ectopy have a markedly increased risk of incident AF. Among the 9.1% of the population with both elevated NT-pro-BNP and frequent SVEs, the absolute risk of incident AF was almost 5% per year, and by the end of follow-up more than half of the subjects in this group had been diagnosed with atrial fibrillation. This risk was similar regardless of CHARGE-AF score. Future studies of primary prevention of AF could focus on this population. Patients with AF are at risk of adverse outcomes, such as heart failure, thrombo-embolism and impaired cognitive function, and this results in substantial mortality and morbidity [24]. Earlier detection and prevention could therefore contribute to reduced AF-associated morbidity, mortality, and medical costs [25].

Both SVE and NT-pro-BNP are established predictors of AF. SVEs have been shown to be associated with incident AF in several cohorts sampled from American, European and Asian populations [14–16, 26]. SVT frequency is associated with incidence of AF independently of symptomatic cardiovascular disease in this cohort and the Copenhagen Holter Study [14, 16]. It is plausible that frequent supraventricular ectopic activity is a marker of existing electrical remodeling, a component of the emerging concept of atrial myopathy which is also characterized by structural remodeling [27]. Structural remodeling is characterized by atrial dilatation, as well as atrial strain [7], and these changes increase the secretion of NT-proBNP from atrium [6, 28–31]. Tissue fibrosis is another characteristic of structural remodeling; this observed in parallel with myocardial apoptosis or necrosis [32]. Troponin T, which is released during cardiomyocyte damage and could possibly reflect fibrosis, is also related to incident AF [11, 33, 34]. This study found a correlation between supraventricular arrhythmias and plasma biomarkers levels, and that the combination of NT-pro-BNP and frequent SVEs was more common among subjects with elevated CHARGE-score, which supports the idea that atrial myopathy is caused by established AF risk factors, and that intervention against modifiable risk factors may slow the progression from atrial myopathy to AF.

Several risk scores and equations have been developed for the prediction of AF [17]. Extensions of these models include information on blood biomarkers, echocardiographic and electrocardiographic measurements, or genetic variants, beyond the information provided by clinical variables [17]. The present study indicates that a model based on current knowledge of the pathophysiological mechanisms behind AF has the potential to further improve AF prediction, and identify subjects with a markedly increased AF risk. This group could potentially benefit from primary prevention and efforts for early detection of AF in order to prevent stroke.

## Strengths and limitations

There are several strengths of present study. The mean age of the study population is relevant and corresponds to an age at which the incidence of AF is relatively high, but before the onset of the majority of AF cases [35]. The endpoint was retrieved from comprehensive patient registers that cover in- and outpatient hospital diagnoses in Sweden. Only two subjects were lost to follow-up due to emigration from Sweden. The registers do not cover AF patients that are treated in primary care only and it has been estimated that specificity and sensitivity is 93% and 80%, respectively, for an AF diagnosis in these registers [36]. Also, some incident cases with asymptomatic AF may have been missed. However, the high overall cumulative incidence of AF in this study (23.6%) is similar to the life-time risk of AF calculated in other-studies [1, 37], implying that few cases of incident AF have been missed.

The sample size of the population was limited, and in the case of weaker associations this would have resulted in a risk of type II error. The population was oversampled of individuals with insulin resistance and as such, this could be regarded as a population with an increased cardiometabolic risk. However, the results were adjusted for HOMA-IR and results were still statistically significant. There are some unmeasured factors which may lead to residual bias, such as other biomarkers. For example, von Willebrand factor is associated with incident AF [38]. In order to assess the risk of residual confounding, the E value was calculated [39] which estimates how strong any unmeasured confounder must be to remove the significance of the observed main results. The E value for the association between the combination of elevated NT-proBNP with frequent SVEs and AF incidence was 5.06 with the confidence limit of 3.11, suggesting that the observed association could be removed by an unmeasured confounder, which is related to both independent and dependent variable with a relative risk of 5.06, indicating a high robustness of current results.

## Conclusions

We conclude that subjects with both frequent SVEs or SVTs and elevated NT-pro-BNP, are at markedly increased risk of AF. A combination of biomarkers and frequent SVEs or SVTs could improve long-term prediction of incident AF.

## Supplementary Information

**Additional file 1: Supplementary Figure 1**. Individuals included in this research. **Supplementary Figure 2**. Incidence of atrial fibrillation in relation to a combination of elevated NT-proBNP and frequent SVEs. **Supplementary Table 1**. Effect estimates of the association between number of SVEs/SVTs and NT-proBNP/TnT.

## Data Availability

The datasets used and/or analyzed during the current study are available from the corresponding author on reasonable request, or upon request from the data access group of Malmö Diet and Cancer study.

## Abbreviations

AF: Atrial fibrillation
ECG: Electrocardiogram
NT-proBNP: N-terminal pro-brain natriuretic peptide
TnT: Troponin T
SVEs: Supraventricular extrasystoles
SVTs: Supraventricular tachycardias
HOMA-IR: Homeostasis model assessment for insulin resistance
LDL: Low density lipoprotein
CI: Confidence interval
OR: Odds ratio
HR: Hazard ratio
NRI: Net reclassification improvement

## Declarations

### Clinical relevance and future perspective

Those with both frequent supraventricular arrhythmia and elevated NT-proBNP have a high risk of future atrial fibrillation. This group could potentially benefit from primary prevention and efforts for early detection of AF.
