# Supplementary material for: Supraventricular arrhythmia, N-terminal pro-brain natriuretic peptide and troponin T concentration in relation to incidence of atrial fibrillation: a prospective cohort study
Source: BMC Cardiovasc Disord. 2021 Mar 12;21:134. doi: 10.1186/s12872-021-01942-6 (PMC7953799; doi:10.1186/s12872-021-01942-6)
Supplement: Supplementary file 1 — Additional file 1: Supplementary Figure 1. Individuals included in this research. Supplementary Figure 2. Incidence of atrial fibrillation in relation to a combination of elevated NT-proBNP and frequent SVEs. Supplementary Table 1. Effect estimates of the association between number of SVEs/SVTs and NT-proBNP/TnT. [file 12872_2021_1942_MOESM1_ESM.docx]

**Supraventricular Arrhythmia, N-terminal pro-Brain Natriuretic Peptide and Troponin T Concentration in Relation to Incidence of Atrial Fibrillation: a Prospective Cohort Study**

Jun Xiao, Anders P Persson, Gunnar Engström, Linda S.B. Johnson

Malmö Diet and Cancer Study Cohort n=30446

Invitation of random 50% subsample, 1991-1994

Cardiovascular sub-study n=6103

HOMA-IR available for n=5533

Random subsample stratified by HOMA-IR levels

HOMA sub-cohort n=909 (16.43%)

Random sample, based on screening day

24hECG screened individuals n=388 (42.68%)

Known AF n=6

Inadequate recordings n=5

Missing NT-proBNP / TnT n=4

Model 1

n=373

Missing covariate n=9

(HOMA-IR score n=7

LDL n=1

Height/weight n=1)

Model 2

n=364

**Supplementary Figure 1. Individuals included in this research.** Model 1: age, sex adjusted. Model 2: Model 1 + smoking status+ anti-hypertension medications + systolic blood pressure + LDL + HOMA score + height + weight

Abbreviations: ECG=electrocardiography; NT-proBNP=N terminal pro B type natriuretic peptide; TnT=troponin T; HOMA-IR= homeostasis model assessment for insulin resistance; LDL= low density lipoprotein.

**
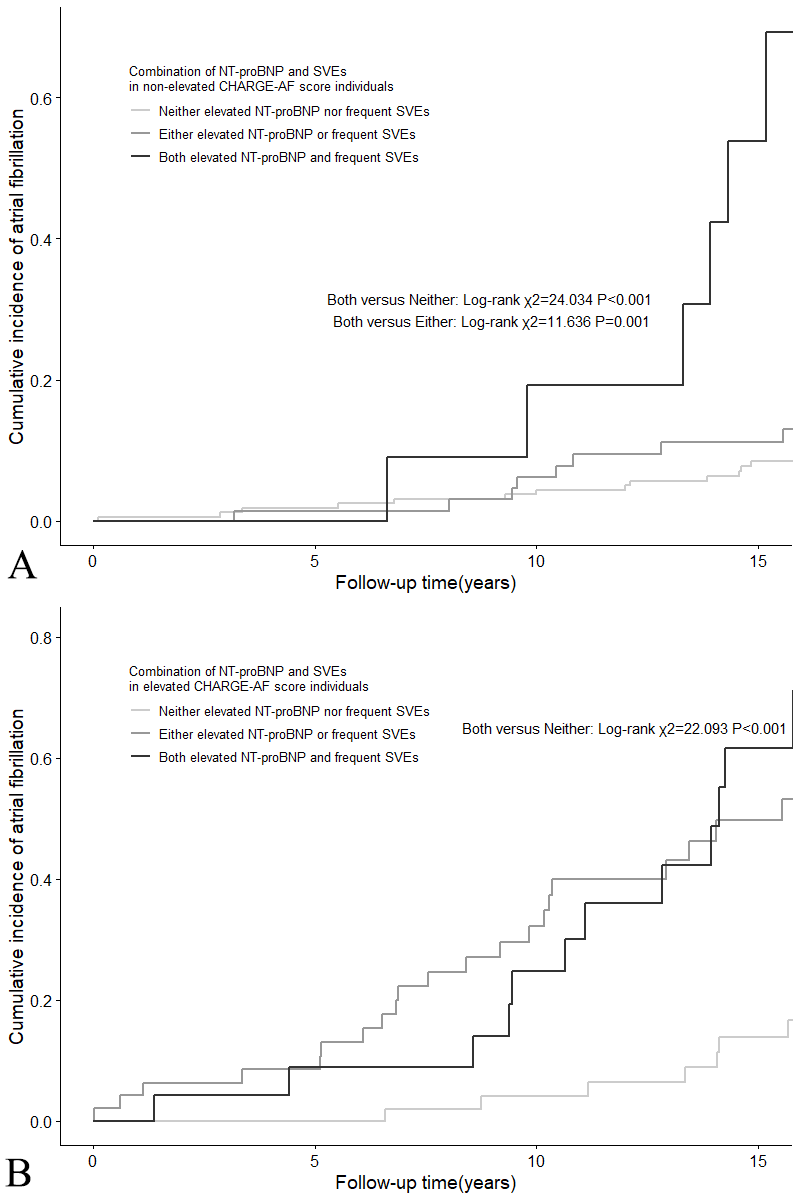
**

**Supplementary Figure 2. Incidence of atrial fibrillation in relation to a combination of elevated NT-proBNP and frequent SVEs. A: in individuals with non-elevated CHARGE-AF score. B: in individuals with elevated CHARGE-AF score.**

Frequent SVEs and elevated NT-proBNP were defined as the top quartile, corresponding to >129.18 SVEs/24 hours and 32.80 pg/ml NT-proBNP, respectively; elevated CHARGE-AF score was defined as top tertile.

Abbreviations: SVEs=supraventricular extrasystoles; NT-proBNP=N terminal pro B type natriuretic peptide.

| **S****upplementary Table 1. Effect estimates of the association between number of SVEs/SVTs and NT-proBNP/TnT** | | | |
| --- | --- | --- | --- |
|  | Crude incidence rate ratio | Model 1 | Model 2 |
| **SVE** |  |  |  |
| NT-proBNP | 1.44(1.19,1.74) | 1.52(1.26,1.82) | **1.64(1.33,2.02)** |
| TnT | 1.16(1.02,1.32) | 1.30(1.12,1.52) | **1.33(1.10,1.60)** |
| **SVT** |  |  |  |
| NT-proBNP | 1.75(1.42,2.16) | 1.73(1.39,2.16) | **1.60(1.27,2.02)** |
| TnT | 1.31(1.11,1.55) | 1.32(1.03,1.71) | **1.29(1.03,1.62)** |

Negative binominal regression was used to estimate the relation between the number of SVEs/SVTs in 24 hours (dependent variables) and per quartile increment in the NT-proBNP and TnT

Model 1: age, sex adjusted (n=373)

Model 2: Model 1 + smoking status+ anti-hypertension medications + systolic blood pressure + LDL + natural log of HOMA-IR +height + weight (n=364)

Abbreviations: NT-proBNP=N terminal pro B type natriuretic peptide; SVEs=supraventricular extrasystoles; SVTs=supraventricular tachycardias; TnT=troponin T.
